# Supplementary material for: The Effect of Dipeptide Repeat Proteins on FUS/TDP43-RNA Condensation in C9orf72 ALS/FTD
Source: J Phys Chem B. 2024 Sep 23;128(39):9405–17. doi: 10.1021/acs.jpcb.4c04663 (PMC11457143; doi:10.1021/acs.jpcb.4c04663)
Supplement: Supplementary file 1 — jp4c04663_si_001.pdf [file jp4c04663_si_001.pdf]

Supplementary Information: The Effect of Dipeptide Repeat Proteins on FUS/TDP43-RNA Condensation in C9orf72 ALS/FTD

M. D. Driver[1], J. Postema[1], and P. R. Onck[1,*]
Zernike Institute for Advanced Materials, University of Groningen, Groningen, 9747AG, The Netherlands
Email: p.r.onck@rug.nl

September 4, 2024

Contents

1 1BPA model **S2**

2 3BPN model **S2**

3 Droplet simulation initialisation **S4**

4 Simulation analysis using a molecular connectivity graph **S5**
4.1 Readouts from a molecular connectivity graph S5
4.2 Contact map definition S6

5 Ternary phase diagram comparison **S7**

6 TDP43-R-DPR and ΔR-DPR binary mixture comparisons **S7**

7 Droplet simulation contact data **S9**

8 Droplet simulation density data **S21**

9 System charge comparison **S22**

1 1BPA model

The 1BPA model was previously developed for the study of the nuclear pore complex (NPC) [1, 2]. It has been extensively used to model the behaviour of the intrinsically disordered nucleoporin regions which fill the center of the NPC and provide a selective barrier to the transport of cargo between the cytoplasm and nucleoplasm [3–12]. In this work we use the updated 1BPA v2.1 developed in [13]. A comparison of the performance of the different versions of the 1BPA model is presented in Fig. S1, with data taken from [13]. The densities of systems computed with the 1BPA-2.1 forcefield (Fig. S22) are comparable to those computed with the Mpipi forcefield [14].

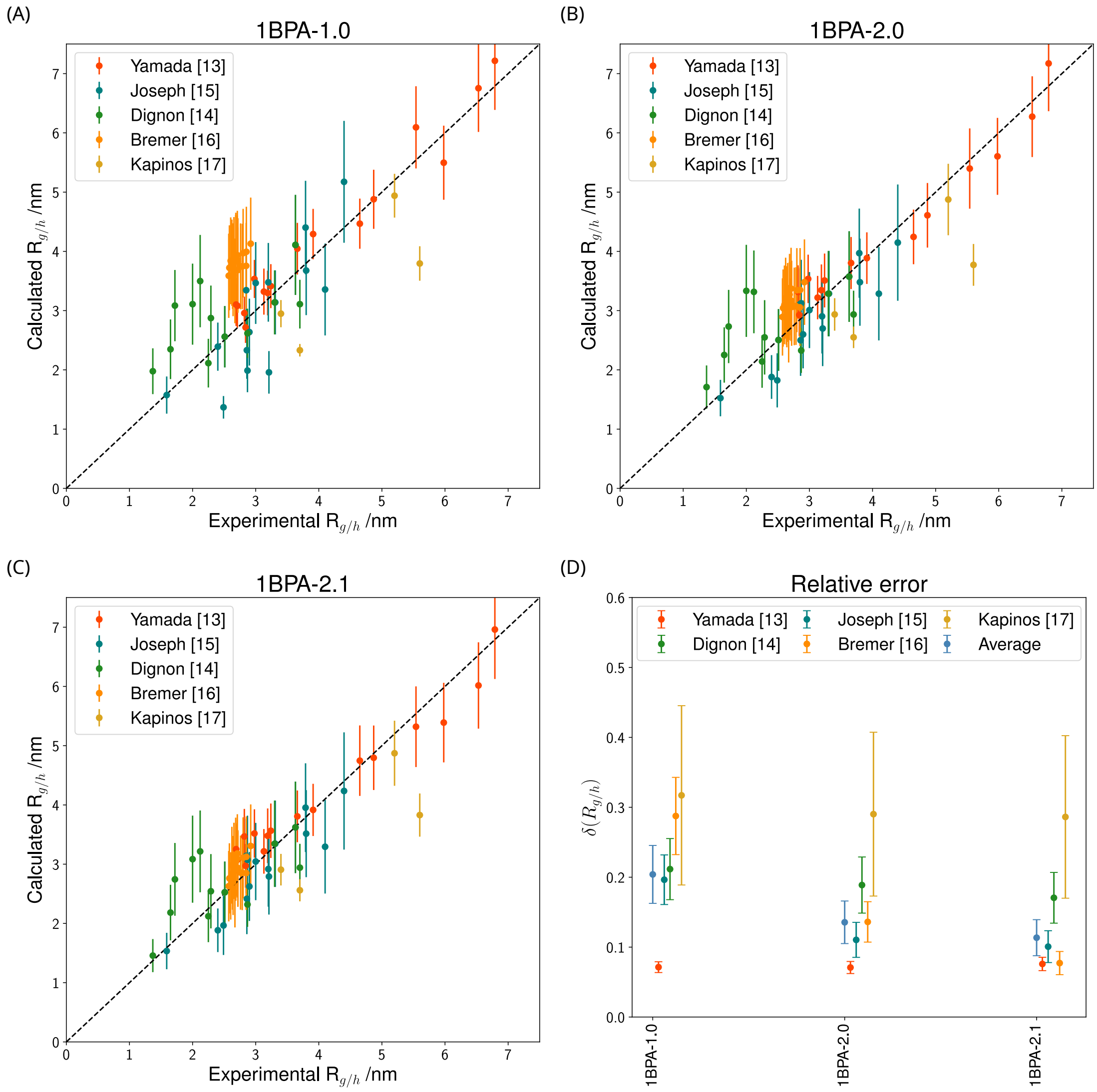

Figure S1: Comparison of computed and experimental radii of gyration, R_g, or hydrodynamic (Stokes) radii, R_h, for (A) version 1BPA-1.0 (from [11]), (B) version 1BPA-2.0 (used in [15, 16]), (C) version 1BPA-2.1 (developed in [13]). (D) $\delta R_{g/h}$, the average relative error between calculated and experimental radii of gyration, R_g, or hydrodynamic (Stokes) radii, R_h, for the datasets shown in (A)-(C). Each dataset is plotted with a small offset for increased clarity. The average dataset is the average of $\delta R_{g/h}$ for the combination of all molecules.

2 3BPN model

The 3 bead per nucleotide (3BPN) model was designed to provide a 1BPA compatible model to be able to simulate mixtures of IDPs and ssRNA. It is based on the three interaction site model of RNA [17].

The 3BPN bonded potential, ϕ_b, is composed of bonds ϕ_{bond} and bending angles ϕ_{bend}, which both take the form of simple harmonic potentials:

$$\phi_b = \phi_{bond} + \phi_{bend}, \tag{1}$$

with ϕ_{bond} and ϕ_{bend} given by,

$$\phi_{\text{bond}} = k_b(r - r_0)^2, \quad (2)$$

$$\phi_{\text{bend}} = k_\theta(\theta - \theta_0)^2, \quad (3)$$

with r_0 and k_b given in Table S1 and θ_0 and k_θ given in Table S2.

Bonds	r_0 /nm	k_b / kJ mol^{-1}nm^{-2}
NB_i-NR_i	0.586	10000
NP_i-NR_i	0.390	10000
NP_i-NR_{i+1}	0.390	10000

Table S1: Bonded parameters where NB_i is the nucleobase bead of nucleotide i (representing either G, C, A or U), NR_i is the ribose bead of nucleotide i and NP_i is the phosphate bead of nucleotide i. Values for r_0 and k_b are taken from [17].

Angles	θ_0 / rad	k_θ / kJ mol^{-1}rad^{-2}
NB_i-NR_i-NP_i	1.76	1000
NP_i-NR_{i+1}-NB_{i+1}	1.59	1000
NR_i-NP_i-NR_{i+1}	1.86	1000
NP_{i-1}-NR_i-NP_i	1.97	1000

Table S2: Angle parameters where NB_i is a nucleotide bead of nucleotide i (representing either G, C, A or U), NR_i is a ribose bead of nucleotide i, NP_i is a phosphate bead of nucleotide i. Values of θ_0 and k_θ are taken from [17].

There are three components that constitute the non-bonded potential, ϕ_{nb} (equation (4)), of the 3BPN model: hydrophobic interactions (ϕ_{hp}, equation (5)), base stacking interactions (ϕ_{bs}, equation (6)) and electrostatic interactions (ϕ_{el}, equation (7)). The hydrophobic (equation (5)) and electrostatic terms (equation (7)) are equivalent to the 1BPA hydrophobic and electrostatic terms to ensure cross compatibility between the two models and enable the simulation of protein-RNA mixtures. Also the ϕ_{bs} has been adjusted to have a similar funtional form as the 1BPA hydrophobicity potential.

$$\phi_{nb} = \phi_{hp} + \phi_{bs} + \phi_{el}, \quad (4)$$

$$\phi_{hp} = \begin{cases} \epsilon_{rep}(\frac{\sigma}{r})^8 - \epsilon_{ij}[\frac{4}{3}(\frac{\sigma}{r})^6 - \frac{1}{3}], & r \leq \sigma \\ \epsilon_{rep} - \epsilon_{ij}(\frac{\sigma}{r})^8 & \sigma \leq r, \end{cases} \quad (5)$$

where $\epsilon_{ij} = \epsilon_{hp}\sqrt{(\epsilon_i\epsilon_j)^\alpha}$, $\sigma = 0.6$ nm, $\epsilon_{rep} = 5$ kJ mol^{-1}, $\alpha = 0.15$, $\epsilon_{hp} = 6.5$ kJ mol^{-1} and ϵ_i, ϵ_j are bead specific hydrophobicities. These were determined by using the Kapcha-Rossky hydrophobicity scale [18] with the 1BPA amino acid hydrophobicities [1, 2], to create a mapping for the nucleotide beads between the Kapcha-Rossky hydrophobicity and the corresponding 1BPA hydrophobicity, see Fig. S2 and the ϵ_i are given in Table S3.

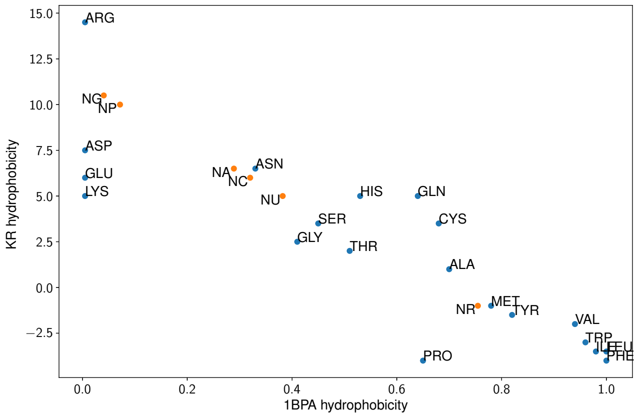

Figure S2: Hydrophobicity scale comparison for the Kapcha-Rossky hydrophobicity scale [18] with the 1BPA amino acid hydrophobicities [1, 2]. Amino acid hydrophobicities are shown by blue dots with the three letter amino acid name. A linear mapping was used to determine the corresponding nucleotide bead 1BPA hydrophobicities (orange dots, labelled by bead name) after computing the Kapcha-Rossky hydrophobicity.

Nucleotide bead	ϵ_i
NP	0.071
NR	0.755
NA	0.289
NC	0.320
NG	0.040
NU	0.382

Table S3: ϵ_i values for 3BPN nucleic acid beads.

$$\phi_{bs} = \begin{cases} \epsilon_{bs1}(\frac{\sigma}{r})^8 - \epsilon_{bs2}[\frac{4}{3}(\frac{\sigma}{r})^6 - \frac{1}{3}], & r \leq \sigma \\ \epsilon_{bs1} - \epsilon_{bs2}(\frac{\sigma}{r})^8 & \sigma \leq r, \end{cases} \tag{6}$$

where $\sigma = 0.6$ nm, $\epsilon_{bs1} = 2.71$ kJ mol^{-1} and $\epsilon_{bs2} = 1.25$ kJ mol^{-1}.

$$\phi_{el} = \frac{q_i q_j}{4\pi\epsilon_0\epsilon_r(r)r} exp(-\kappa r), \tag{7}$$

where $\epsilon_r(r) = S_s[1 - \frac{r^2}{z^2}\frac{e^{r/z}}{(e^{r/z}-1)^2}$, $S_s = 80$ and $z = 0.25$ nm. κ is the Debye screening coefficient which is a function of temperature and ion concentration.

3 Droplet simulation initialisation

All molecules are placed into the initial box which is partitioned into a grid of voxels. The number of voxels is sufficiently large to contain all molecules. Each voxel is sufficiently large to contain the largest molecule without overlap with adjacent voxels, with an additional spacing of 0.6 nm between voxels. A random molecule is assigned to each voxel (note that a voxel may be empty if the total number of molecules is not a cubic number), ensuring a random starting configuration as shown by Fig. S3.

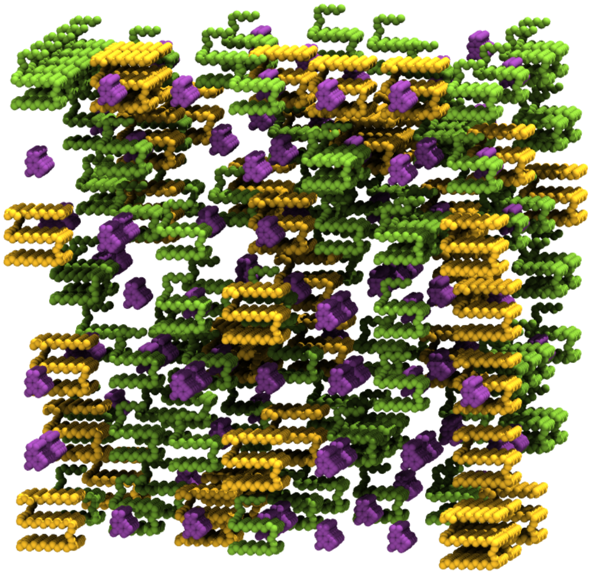

Figure S3: **Starting configuration for the simulation of 40 FUS, 89 U_{40}, 89 PR_{60} at 150 mM ion concentration and 300 K.** FUS molecules are coloured in yellow, U_{40} molecules are coloured in purple, and polyPR_{60} molecules are coloured in green. This corresponds to the centre at (33.3, 33.3, 33.3) on the ternary FUS-U_{40}-PR_{60}diagram.

4 Simulation analysis using a molecular connectivity graph

To assess condensate stability in the droplet simulations, a molecular connectivity graph, consisting of nodes and edges, is used to describe the clustering and assess the time evolution of the molecular network during the simulation. Each simulation frame can be described by a connectivity graph. In the graph representation each node corresponds to a unique molecule, with an edge between the nodes denoting the number of non-zero interactions (if zero interactions are present no edge is added). Within an edge information can be stored about the number and type of contacts between molecules, based on residue categorisation. An interaction was determined to be present using a cutoff of 0.7 nm between 1BPA residues [3]. The cutoff of 0.7 nm represents the bead diameter (0.6 nm) plus an additional 0.1 nm buffer to account for thermal fluctuations to ensure a convergence in contacts. This was previously explored in Ghavami *et al.* [3]. The relative importance of the contributions of different interactions is a function of the choice of cutoff distance used. The aromatic interactions are more prominent at a cutoff distance of 0.6 nm (Fig. S9 in the SI), due to a smaller equilibrium distance for these interactions. The importance of cation-π interactions, which have on average a larger equilibrium distance only become prominent with a cutoff of 0.7 nm (Fig. 2). At cutoffs above 0.7 nm, convergence in the contact maps was observed (See Figs. S10 and S11 in the SI for 0.8 nm and 1.0 nm cutoffs respectively), albeit with lower numbers of contacts.

Computation and processing of the contact matrix for a simulation frame allows the creation of the molecule graph. This process is shown in figure S4.

4.1 Readouts from a molecular connectivity graph

A molecular connectivity graph computed for a simulation can be used to get several different readouts about a simulation system. Information on the size of clusters can be found by identifying the discrete sub-graphs of nodes with only internal connections (no contacts outside of the cluster). Through assessment of the time evolution of the number of clusters, grouped based on number of molecules, and the number of molecules in clusters within specific size categories it is possible to determine whether the system has reached equilibrium. After convergence is established it is possible to examine the distribution of components within the different clusters. The percentage of species i in a cluster is computed using $N_{i \in C}/(N_{frames}N_i)$ where $N_{i \in C}$ is the number of molecules of i in a cluster of size C.

In addition to collating node information from a cluster, the number and type of interactions stored in the graph edges can also be used to assess the simulations. Interaction data can be aggregated based on the type of species involved. Normalisation is again required to be able to compare the relative number of interactions between molecules, using $N_iL_iN_jL_j$, where L_i and L_j are the number of simulation residues in molecules i and j, respectively.

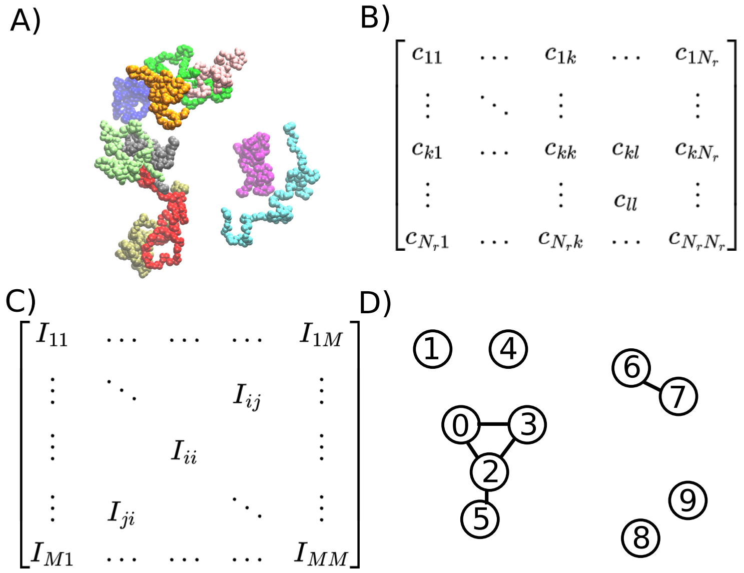

Figure S4: Processing of a contact matrix for one trajectory frame. (A) Visualisation of molecules in the trajectory frame. The particles of the 10 molecules are coloured based on the molecule type. (B) The contact matrix for a frame is computed between all residues, yielding an $N_r \times N_r$ matrix, with a value of 1 if a contact is present between particles k and l, and 0 otherwise. (C) The contact matrix in (B) can be decomposed into distinct sub-matrices by grouping together residue interactions belonging to unique molecule pairs. This produces a new matrix representation, where element I_{ij} is the contact matrix between molecules i and j. (D) A molecular graph derived from the contact matrix in (C). When constructing the molecular graph we consider only the elements where $i \geq j$ to avoid double counting of interactions. We iterate over the I_{ij} matrices ($i \neq j$), to add edges to the graph, storing information about the number of intermolecular contacts between molecules i and j. Similarly, a summation of intramolecular contacts I_{ii} for like molecules (case when $i = j$) is also stored. This process is repeated for all frames in a simulation with a sampling rate of once every 50 ns.

4.2 Contact map definition

From the system graph creation process, contact matrices for all molecule combinations, over all frames are computed. This information is also useful for understanding the specific residues which drive condensation. As such, this information is aggregated during graph computation. Contact maps can be divided into two categories: intramolecular contact maps (interactions between particles in the same molecule copy), corresponding to the diagonal sub-matrices (I_{ii}) in figure S4, and intermolecular contact maps (interactions between particles of two different molecules), corresponding to the off-diagonal sub-matrices (I_{ij}) in figure S4. On the macroscale individual molecules of the same type are indistinguishable, thus only unique combinations of molecules need to be stored in different matrices. A system has M intramolecular contact maps and $M(M+1)/2$ intermolecular contact maps where M is the number of distinguishable species. Aggregation over all frames and molecule copies of the contact maps is therefore possible for memory efficient storage during computation. To display the information stored in the contact map as a contact probability, ordered by particle index in a molecule, the contact information is normalised by the number of frames and $((N_i N_j)^2)$ where N_i and N_j are the number of copies of molecule types i and j in the simulation. This is to allow comparison between species in the simulation where the number of copies may be very large. To display the information based on residue type a matrix reduction is used on the contact map plotted by residue index to sum interactions of particles with the same type. Further normalisation can be undertaken of this matrix to normalise the interactions by $(N_{i,r1} N_{j,r2})^2$, with $N_{j,r1}$ the number of residue $r1$ in molecule i and $N_{j,r2}$ the number of residue $r2$ in molecule j.

5 Ternary phase diagram comparison

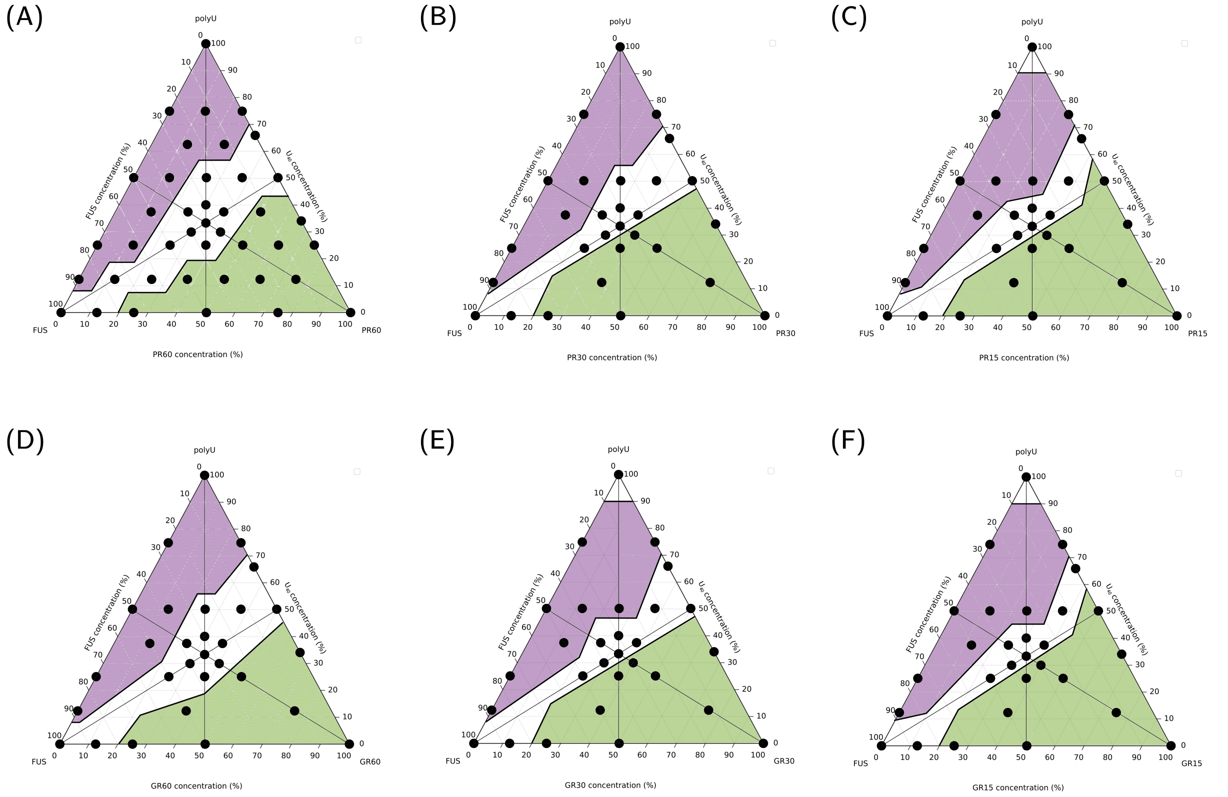

Figure S5: Ternary phase diagrams for (A) PR_{60}, (B) PR_{30}, (C) PR_{15}, (D) GR_{60}, (E) GR_{30} and (F) GR_{15}. Triangles showing summary of phases found to highlight any changes- these are currently in SI for discussion. There is a small change to the two boundaries narrowing on the centre region with R-DPR length.

6 TDP43-R-DPR and ΔR-DPR binary mixture comparisons

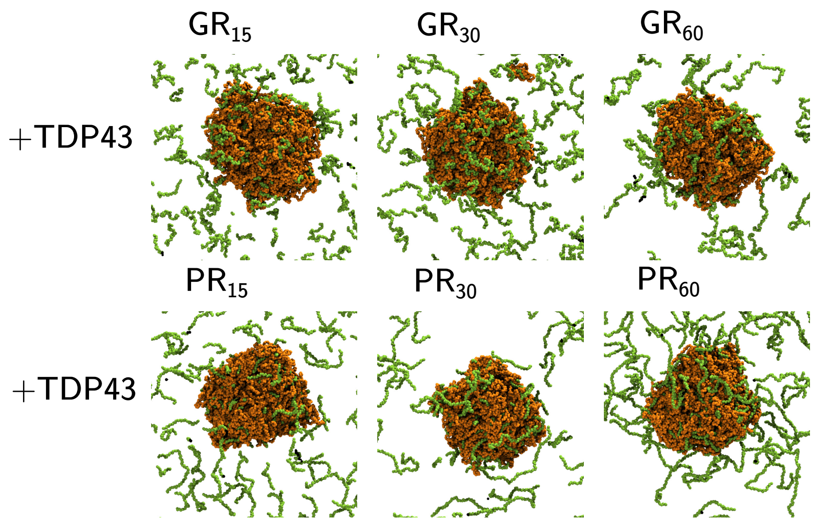

Figure S6: Figure showing the behaviour of TDP43 with different R-DPR sequences. All simulations are done at equal fraction DPR to TDP43.

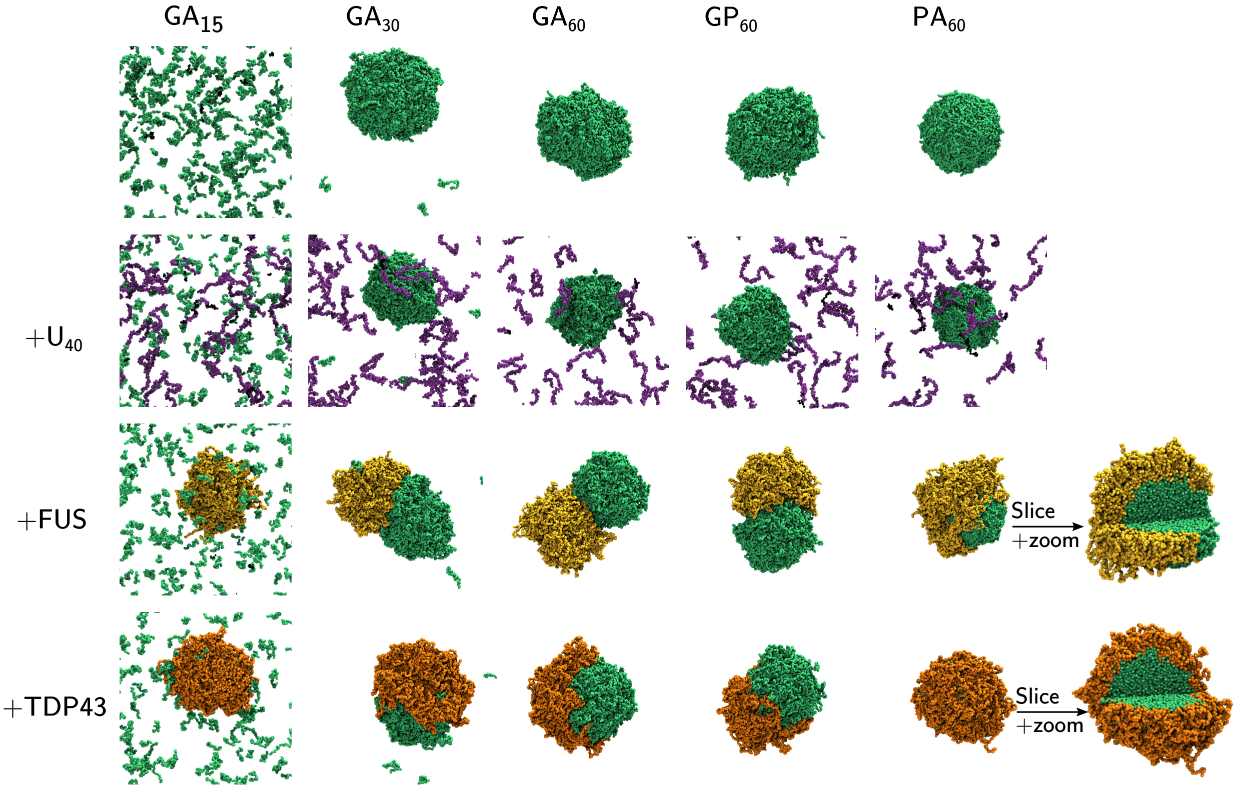

Figure S7: PS behaviour of ΔR-DPR sequences in isolation and with various partners. Top row isolated ΔR-DPR, second row ΔR-DPR with U_{40}, third row ΔR-DPR with FUS, fourth row ΔR-DPR with TDP-43. All simulations are done at an equal fraction of DPR to U_{40}/FUS/TDP43.

7 Droplet simulation contact data

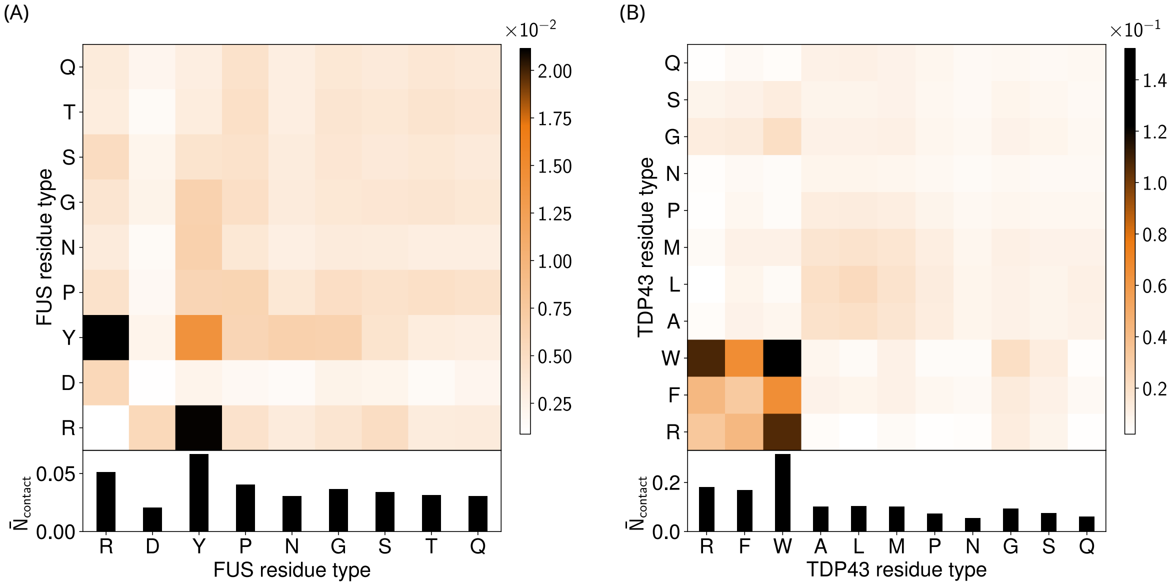

Figure S8: **Normalised homotypic intermolecular contact maps.** (A) 100% FUS and (B) 100% TDP43 at 150 mM and 300 K. For the definitions of the normalised contact types see section 4.

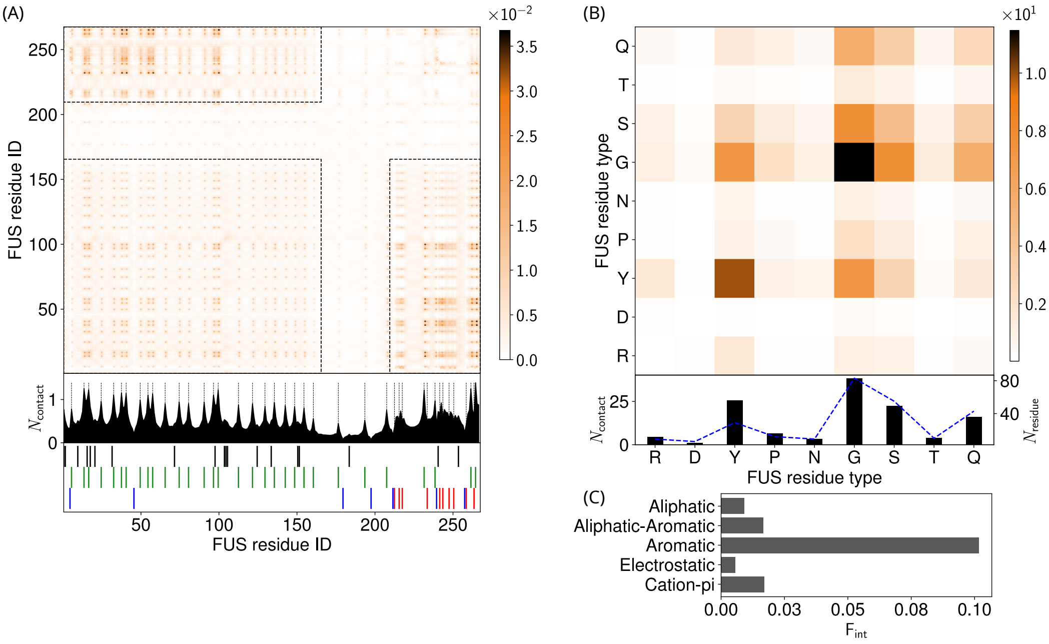

Figure S9: **Intermolecular contact information for FUS interactions at 0.6 nm cutoff.** (A) FUS intermolecular contact map by residue index for 100% FUS at 150 mM ion concentration and 300 K. The contacts are averaged in time and normalised by the number of molecules in the simulation (see section 4.2 in the SI for more details). A 1D contact profile (summation of the 2D map) is included below the contact map to show the total interactions per residue index (N_{contact}). The black dashed lines highlight the location of tyrosine and arginine residues (which correspond to the peaks in the 1D profile). (B) Intermolecular contact map by residue type for FUS. The contact map in (B) is similar to the contact map by residue index in (A), but aggregated by residue type. A 1D contact profile (summation of the 2D map), are also included below (N_{contact}) together with the abundance for the residues (N_{residue}) shown by blue dashed lines. (C) Interaction summary for a droplet simulation with 100% FUS at 150 mM and 300 K. The fraction of interactions, F_{int}, are aggregated by type and normalised by the total number of interactions. Aromatic and aliphatic interactions denote aromatic-aromatic and aliphatic-aliphatic interactions respectively. This convention is used throughout this work. Details of the contact definitions can be found in section 4.2.

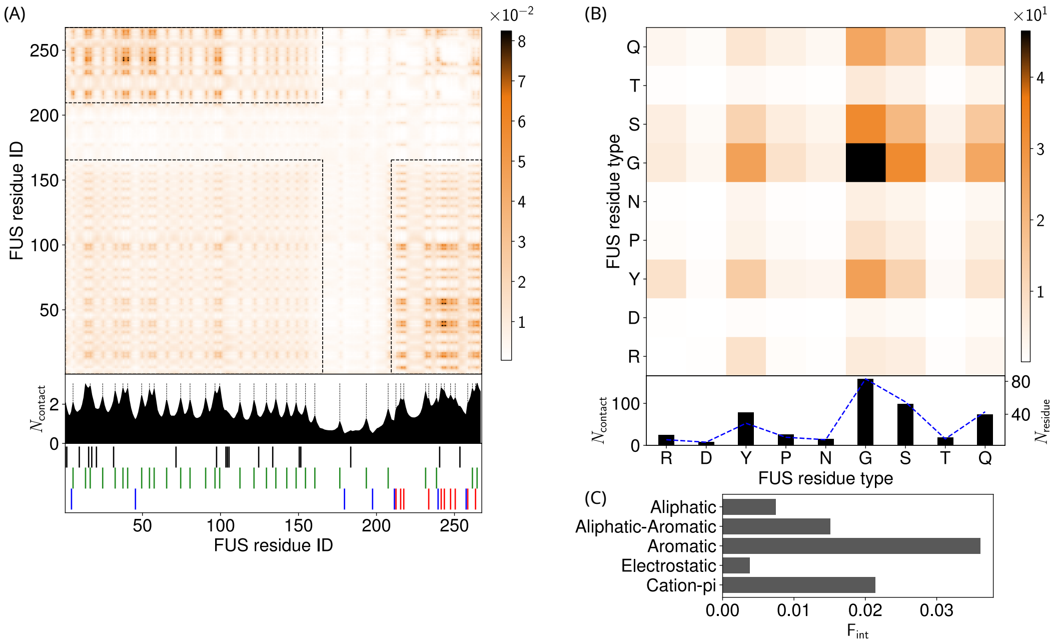

Figure S10: **Intermolecular contact information for FUS interactions at 0.8 nm cutoff.** (A) FUS intermolecular contact map by residue index for 100% FUS at 150 mM ion concentration and 300 K. The contacts are averaged in time and normalised by the number of molecules in the simulation (see section 4.2 in the SI for more details). A 1D contact profile (summation of the 2D map) is included below the contact map to show the total interactions per residue index (N_{contact}). The black dashed lines highlight the location of tyrosine and arginine residues (which correspond to the peaks in the 1D profile). (B) Intermolecular contact map by residue type for FUS. The contact map in (B) is similar to the contact map by residue index in (A), but aggregated by residue type. A 1D contact profile (summation of the 2D map), are also included below (N_{contact}) together with the abundance for the residues (N_{residue}) shown by blue dashed lines. (C) Interaction summary for a droplet simulation with 100% FUS at 150 mM and 300 K. The fraction of interactions, $\mathsf{F}_{\mathsf{int}}$, are aggregated by type and normalised by the total number of interactions. Aromatic and aliphatic interactions denote aromatic-aromatic and aliphatic-aliphatic interactions respectively. This convention is used throughout this work. Details of the contact definitions can be found in section 4.2.

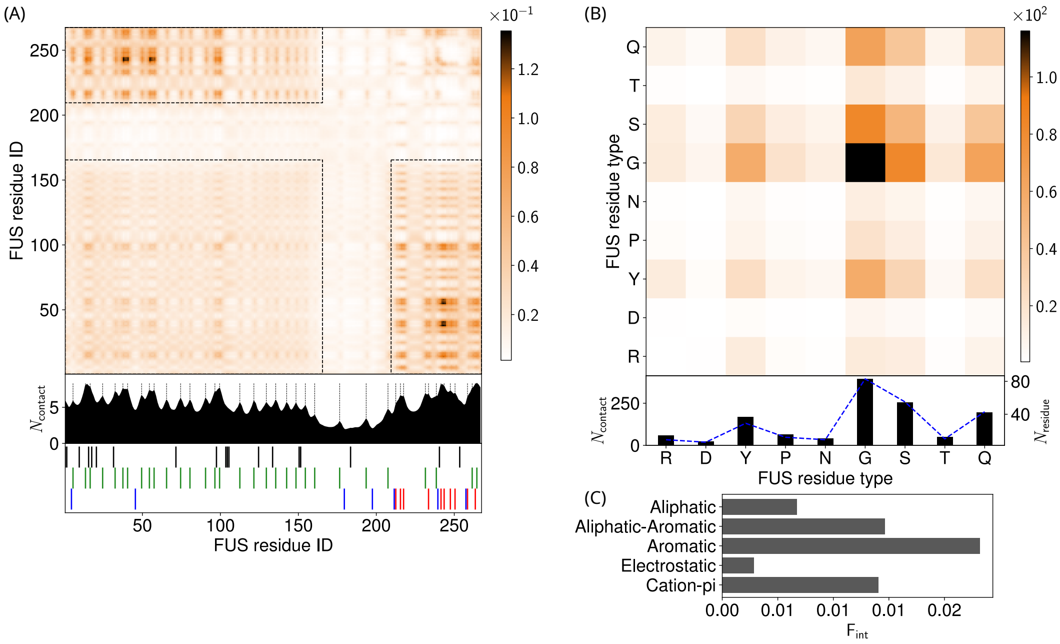

Figure S11: **Intermolecular contact information for FUS interactions at 1.0 nm cutoff.** (A) FUS intermolecular contact map by residue index for 100% FUS at 150 mM ion concentration and 300 K. The contacts are averaged in time and normalised by the number of molecules in the simulation (see section 4.2 in the SI for more details). A 1D contact profile (summation of the 2D map) is included below the contact map to show the total interactions per residue index (N_{contact}). The black dashed lines highlight the location of tyrosine and arginine residues (which correspond to the peaks in the 1D profile). (B) Intermolecular contact map by residue type for FUS. The contact map in (B) is similar to the contact map by residue index in (A), but aggregated by residue type. A 1D contact profile (summation of the 2D map), are also included below (N_{contact}) together with the abundance for the residues (N_{residue}) shown by blue dashed lines. (C) Interaction summary for a droplet simulation with 100% FUS at 150 mM and 300 K. The fraction of interactions, $\mathsf{F}_{\mathsf{int}}$, are aggregated by type and normalised by the total number of interactions. Aromatic and aliphatic interactions denote aromatic-aromatic and aliphatic-aliphatic interactions respectively. This convention is used throughout this work. Details of the contact definitions can be found in section 4.2.

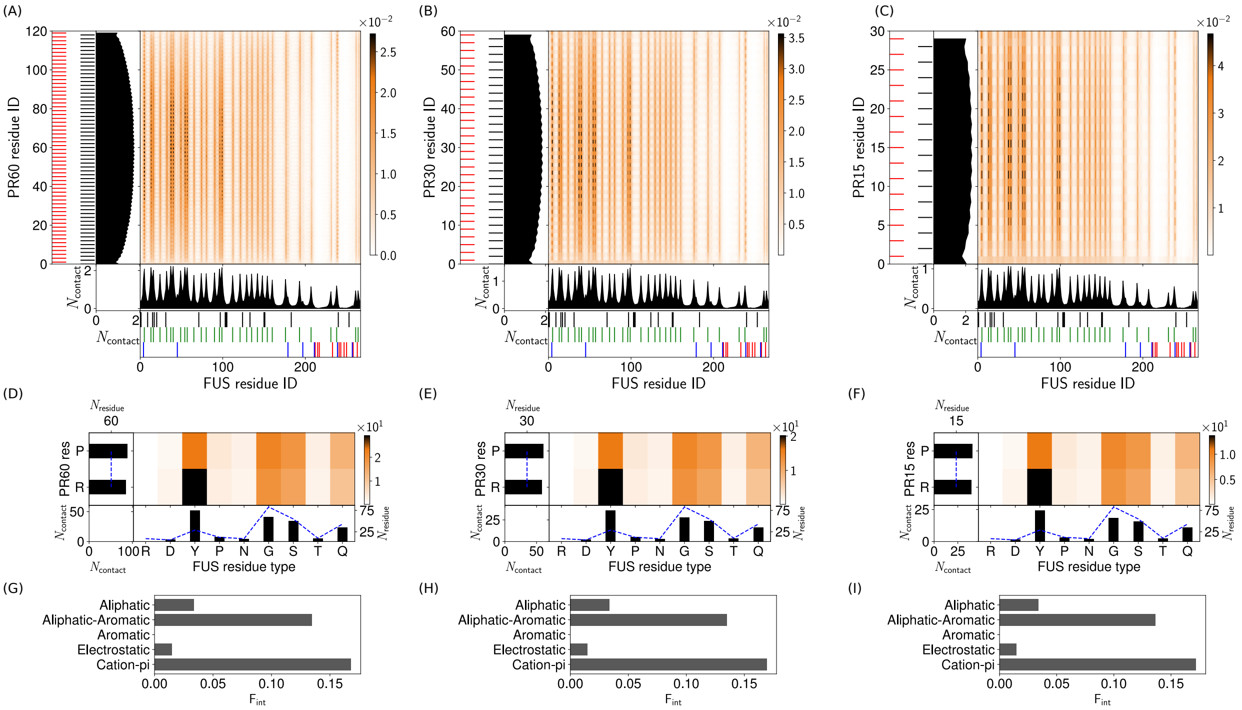

Figure S12: **Intermolecular contact maps for heterotypic interactions in two-component droplets.** (A)-(C) Intermolecular contact map by residue index for (A) FUS with PR60 (50%, 50%), (B) FUS with PR30 (50%, 50%), and (C) FUS with PR15 (50%, 50%) at 150 mM and 300 K. For the definitions of the different contact types see section 4. The 1D contact profiles denote a summation of the 2D map of the corresponding molecules. The black dashed lines highlight the key residues: the aromatic residues in FUS. (D)-(F) Intermolecular contact map by residue type for (D) FUS with PR60 (50%, 50%), (E) FUS with PR30 (50%, 50%), and (F) FUS with PR15 (50%, 50%) at 150 mM and 300 K. (G)-(I) Intermolecular interaction summary for (G) FUS-PR60 interactions in (50%, 50%), (H) FUS-PR30 interactions in (50%, 50%), and (I) FUS-PR15 interactions in (50%, 50%) at 150 mM and 300 K. The fraction of interactions, $\mathsf{F}_{\mathsf{int}}$, are aggregated by type and normalised by the total number of the intermolecular interactions in (A)-(C) respectively.

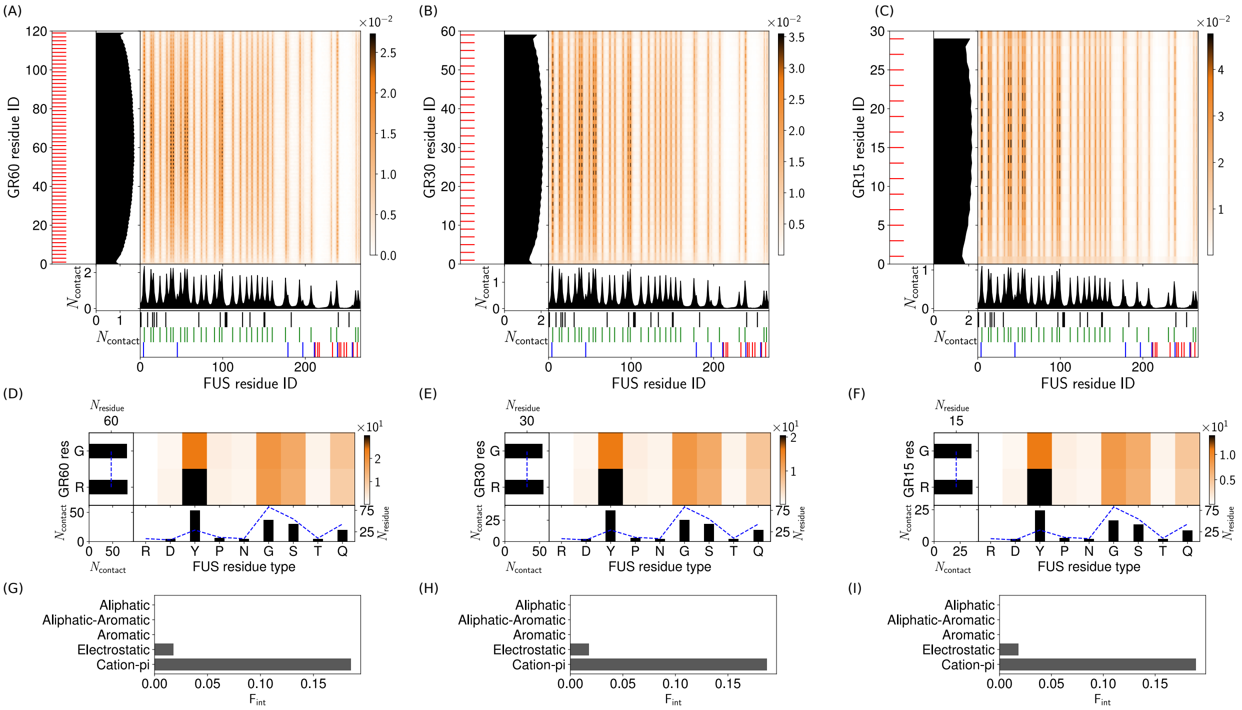

Figure S13: **Intermolecular contact maps for heterotypic interactions in two-component droplets.** (A)-(C) Intermolecular contact map by residue index for (A) FUS with GR60 (50%, 50%), (B) FUS with GR30 (50%, 50%), and (C) FUS with GR15 (50%, 50%) at 150 mM and 300 K. For the definitions of the different contact types see section 4. The 1D contact profiles denote a summation of the 2D map of the corresponding molecules. The black dashed lines highlight the key residues: the aromatic residues in FUS. (D)-(F) Intermolecular contact map by residue type for (D) FUS with GR60 (50%, 50%), (E) FUS with GR30 (50%, 50%), and (F) FUS with GR15 (50%, 50%) at 150 mM and 300 K. (G)-(I) Intermolecular interaction summary for (G) FUS-GR60 interactions in (50%, 50%), (H) FUS-GR30 interactions in (50%, 50%), and (I) FUS-GR15 interactions in (50%, 50%) at 150 mM and 300 K. The fraction of interactions, F_{int}, are aggregated by type and normalised by the total number of the intermolecular interactions in (A)-(C) respectively.

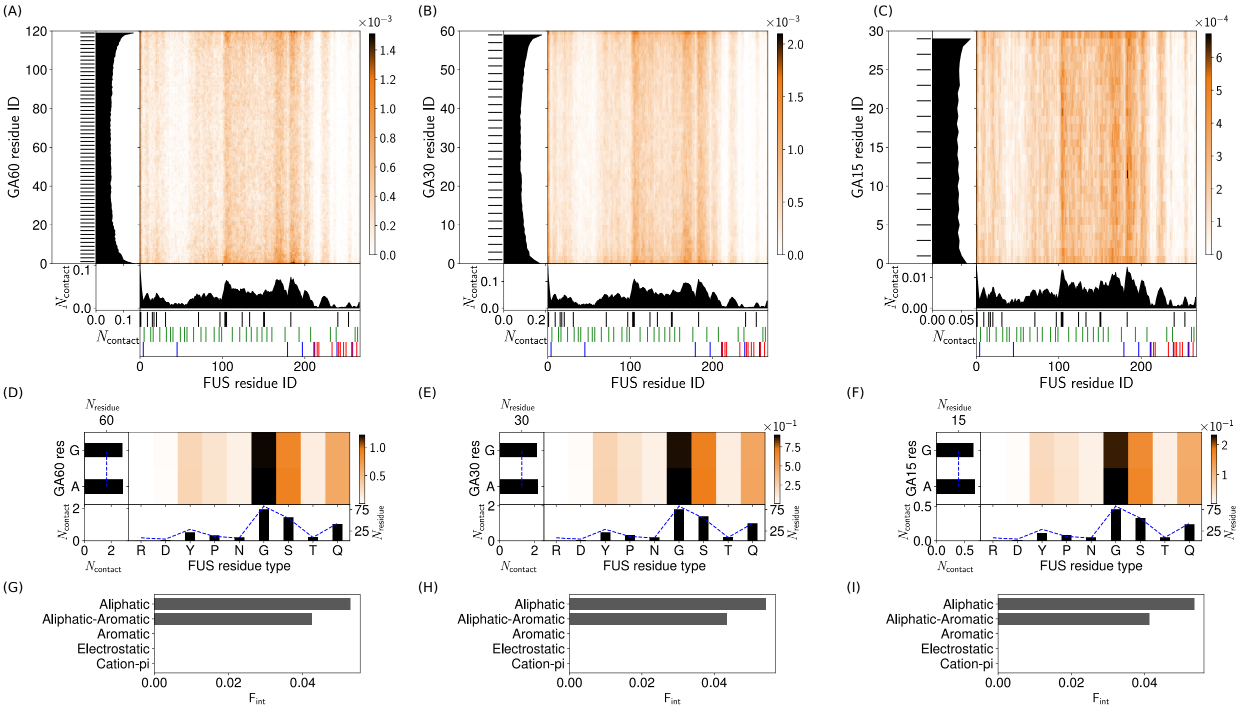

Figure S14: **Intermolecular contact maps for heterotypic interactions in two-component droplets.** (A)-(C) Intermolecular contact map by residue index for (A) FUS with GA60 (50%, 50%), (B) FUS with GA30 (50%, 50%), and (C) FUS with GA15 (50%, 50%) at 150 mM and 300 K. For the definitions of the different contact types see section 4. The 1D contact profiles denote a summation of the 2D map of the corresponding molecules. The black dashed lines highlight the key residues: the aromatic residues in FUS. (D)-(F) Intermolecular contact map by residue type for (D) FUS with GA60 (50%, 50%), (E) FUS with GA30 (50%, 50%), and (F) FUS with GA15 (50%, 50%) at 150 mM and 300 K. (G)-(I) Intermolecular interaction summary for (G) FUS-GA60 interactions in (50%, 50%), (H) FUS-GA30 interactions in (50%, 50%), and (I) FUS-GA15 interactions in (50%, 50%) at 150 mM and 300 K. The fraction of interactions, $\mathsf{F}_{\mathsf{int}}$, are aggregated by type and normalised by the total number of the intermolecular interactions in (A)-(C) respectively.

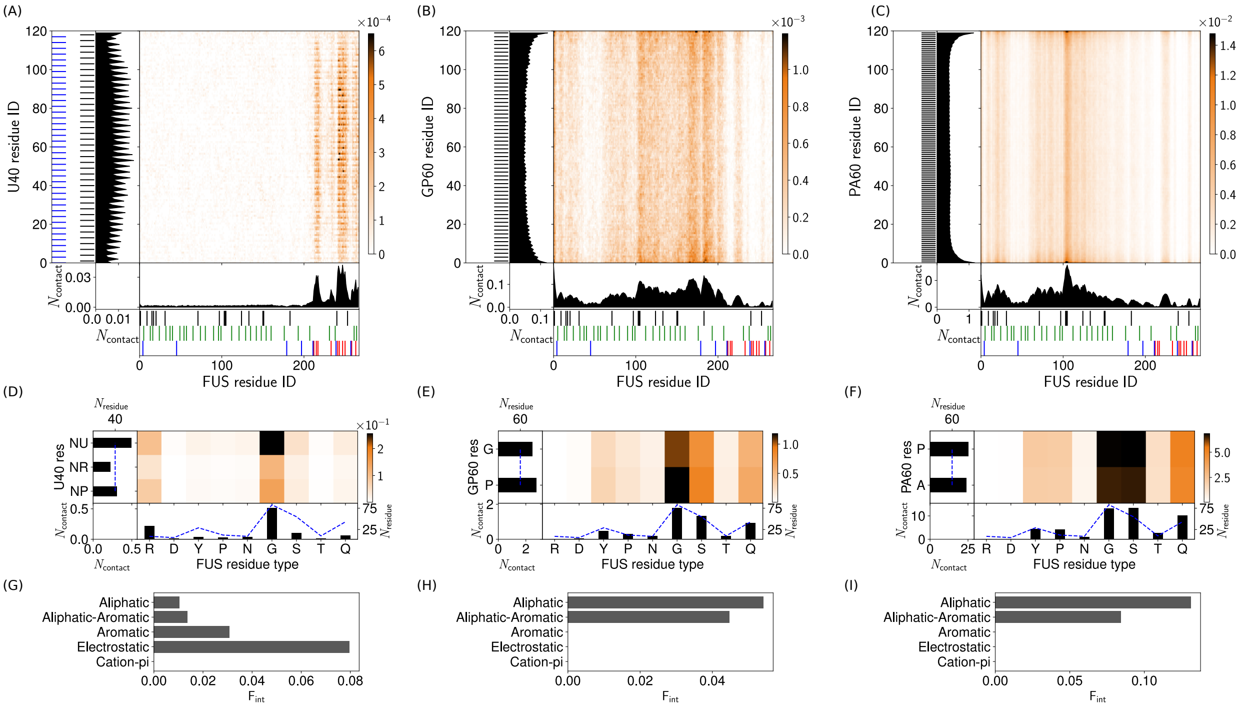

Figure S15: **Intermolecular contact maps for heterotypic interactions in two-component droplets.** (A)-(C) Intermolecular contact map by residue index for (A) FUS with U40 (50%, 50%), (B) FUS with GP60 (50%, 50%), and (C) FUS with PA60 (50%, 50%) at 150 mM and 300 K. For the definitions of the different contact types see section 4. The 1D contact profiles denote a summation of the 2D map of the corresponding molecules. The black dashed lines highlight the key residues: the aromatic residues in FUS. (D)-(F) Intermolecular contact map by residue type for (D) FUS with U40 (50%, 50%), (E) FUS with GP60 (50%, 50%), and (F) FUS with PA60 (50%, 50%) at 150 mM and 300 K. (G)-(I) Intermolecular interaction summary for (G) FUS-U40 interactions in (50%, 50%), (H) FUS-GP60 interactions in (50%, 50%), and (I) FUS-PA60 interactions in (50%, 50%) at 150 mM and 300 K. The fraction of interactions, $\mathsf{F_{int}}$, are aggregated by type and normalised by the total number of the intermolecular interactions in (A)-(C) respectively.

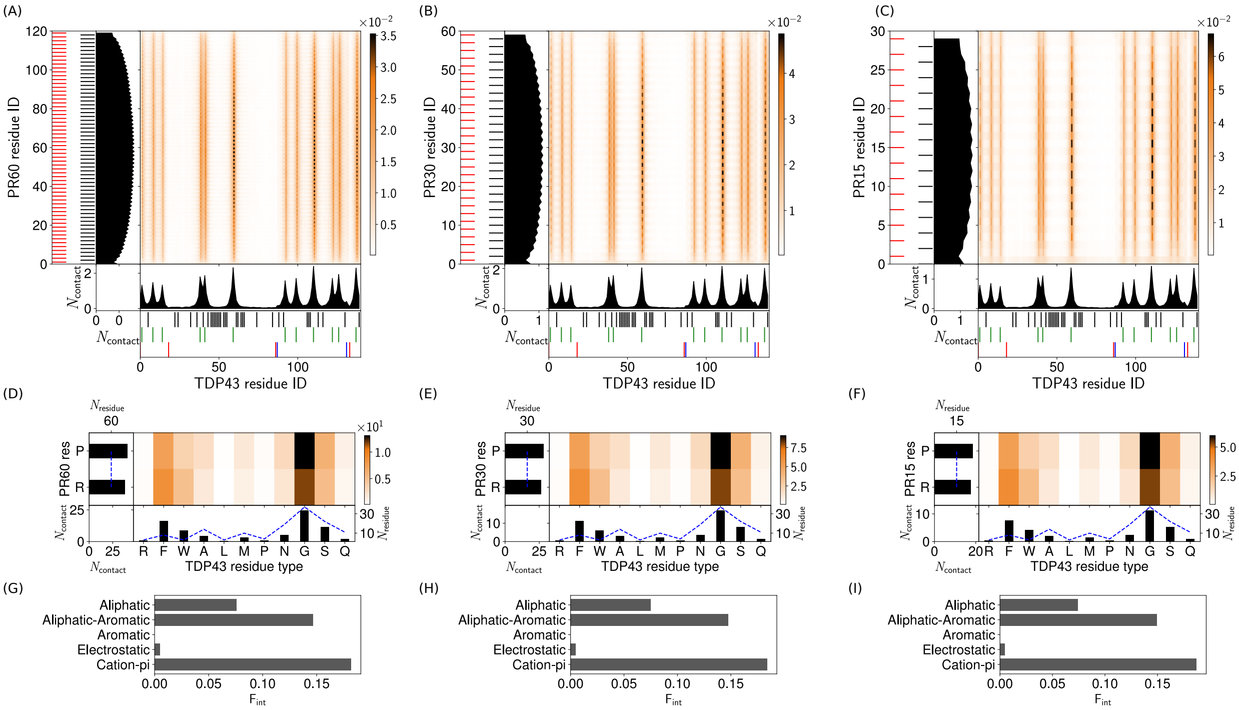

Figure S16: **Intermolecular contact maps for heterotypic interactions in two-component droplets.** (A)-(C) Intermolecular contact map by residue index for (A) TDP43 with PR60 (50%, 50%), (B) TDP43 with PR30 (50%, 50%), and (C) TDP43 with PR15 (50%, 50%) at 150 mM and 300 K. For the definitions of the different contact types see section 4. The 1D contact profiles denote a summation of the 2D map of the corresponding molecules. (D)-(F) Intermolecular contact map by residue type for (D) TDP43 with PR60 (50%, 50%), (E) TDP43 with PR30 (50%, 50%), and (F) TDP43 with PR15 (50%, 50%) at 150 mM and 300 K. (G)-(I) Intermolecular interaction summary for (G) TDP43-PR60 interactions in (50%, 50%), (H) TDP43-PR30 interactions in (50%, 50%), and (I) TDP43-PR15 interactions in (50%, 50%) at 150 mM and 300 K. The fraction of interactions, $\mathsf{F}_{\mathrm{int}}$, are aggregated by type and normalised by the total number of the intermolecular interactions in (A)-(C) respectively.

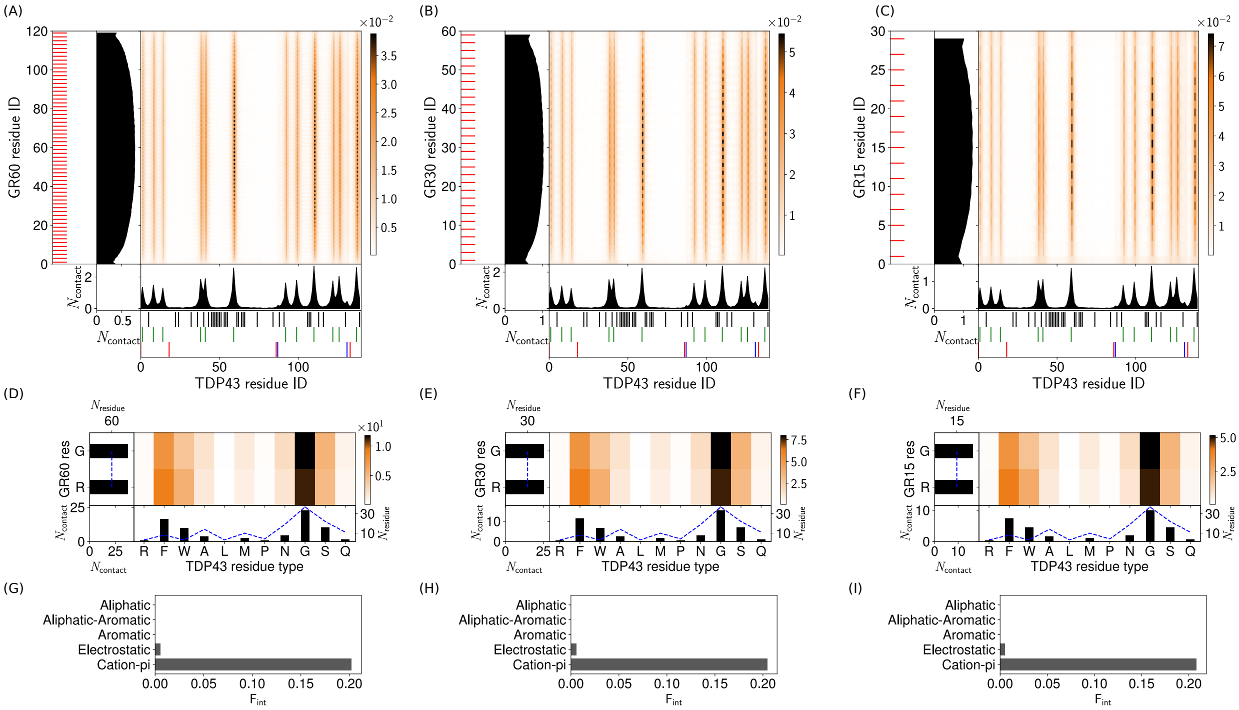

Figure S17: **Intermolecular contact maps for heterotypic interactions in two-component droplets.** (A)-(C) Intermolecular contact map by residue index for (A) TDP43 with GR60 (50%, 50%), (B) TDP43 with GR30 (50%, 50%), and (C) TDP43 with GR15 (50%, 50%) at 150 mM and 300 K. For the definitions of the different contact types see section 4. The 1D contact profiles denote a summation of the 2D map of the corresponding molecules. (D)-(F) Intermolecular contact map by residue type for (D) TDP43 with GR60 (50%, 50%), (E) TDP43 with GR30 (50%, 50%), and (F) TDP43 with GR15 (50%, 50%) at 150 mM and 300 K. (G)-(I) Intermolecular interaction summary for (G) TDP43-GR60 interactions in (50%, 50%), (H) TDP43-GR30 interactions in (50%, 50%), and (I) TDP43-GR15 interactions in (50%, 50%) at 150 mM and 300 K. The fraction of interactions, $\mathsf{F}_{\mathsf{int}}$, are aggregated by type and normalised by the total number of the intermolecular interactions in (A)-(C) respectively.

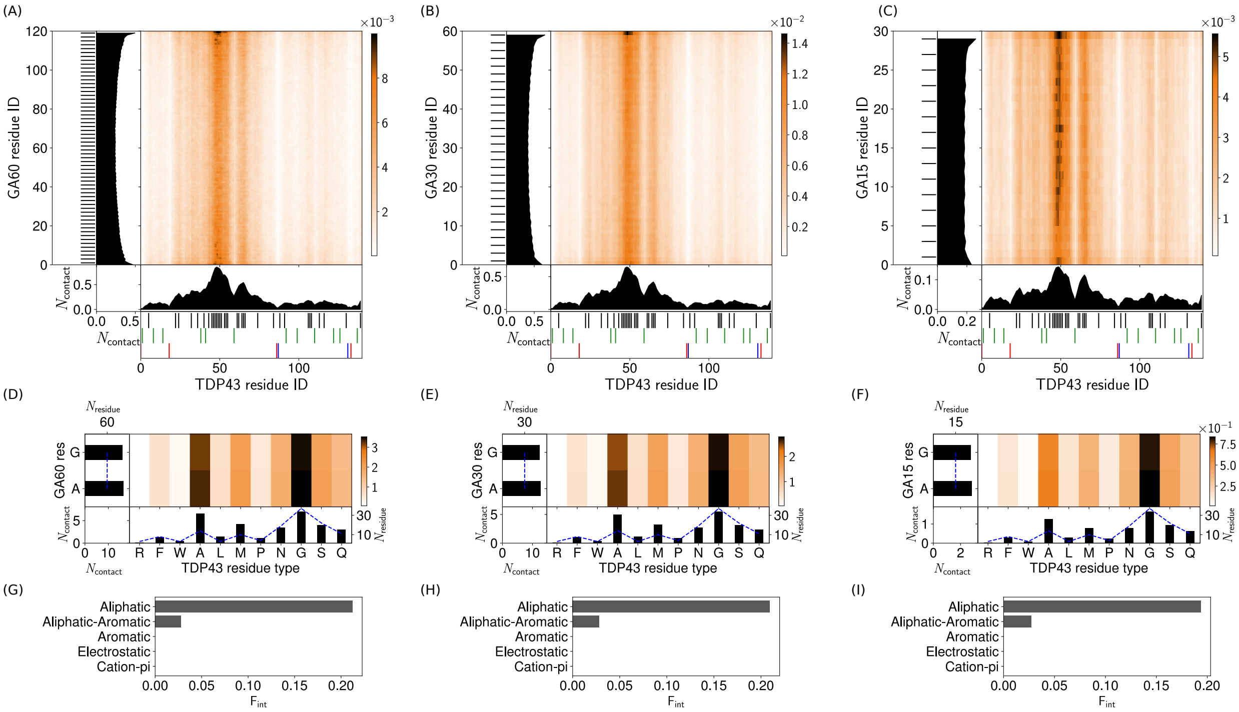

Figure S18: **Intermolecular contact maps for heterotypic interactions in two-component droplets.** (A)-(C) Intermolecular contact map by residue index for (A) TDP43 with GA60 (50%, 50%), (B) TDP43 with GA30 (50%, 50%), and (C) TDP43 with GAR15 (50%, 50%) at 150 mM and 300 K. For the definitions of the different contact types see section 4. The 1D contact profiles denote a summation of the 2D map of the corresponding molecules. (D)-(F) Intermolecular contact map by residue type for (D) TDP43 with GA60 (50%, 50%), (E) TDP43 with GA30 (50%, 50%), and (F) TDP43 with GA15 (50%, 50%) at 150 mM and 300 K. (G)-(I) Intermolecular interaction summary for (G) TDP43-GA60 interactions in (50%, 50%), (H) TDP43-GA30 interactions in (50%, 50%), and (I) TDP43-GA15 interactions in (50%, 50%) at 150 mM and 300 K. The fraction of interactions, $\mathsf{F}_{\mathsf{int}}$, are aggregated by type and normalised by the total number of the intermolecular interactions in (A)-(C) respectively.

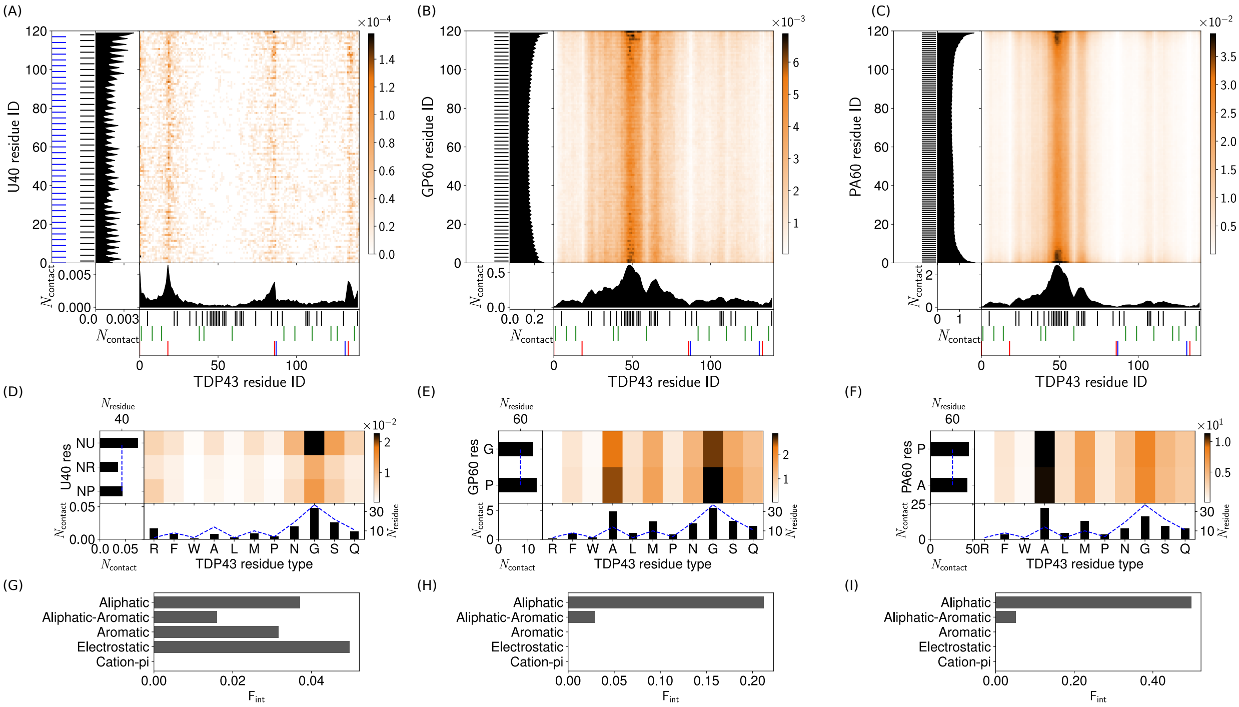

Figure S19: **Intermolecular contact maps for heterotypic interactions in two-component droplets.** (A)-(C) Intermolecular contact map by residue index for (A) TDP43 with U40 (50%, 50%), (B) TDP43 with GP60 (50%, 50%), and (C) TDP43 with PA60 (50%, 50%) at 150 mM and 300 K. For the definitions of the different contact types see section 4. The 1D contact profiles denote a summation of the 2D map of the corresponding molecules. (D)-(F) Intermolecular contact map by residue type for (D) TDP43 with U40 (50%, 50%), (E) TDP43 with GP60 (50%, 50%), and (F) TDP43 with PA60 (50%, 50%) at 150 mM and 300 K. (G)-(I) Intermolecular interaction summary for (G) TDP43-U40 interactions in (50%, 50%), (H) TDP43-GP60 interactions in (50%, 50%), and (I) TDP43-PA60 interactions in (50%, 50%) at 150 mM and 300 K. The fraction of interactions, $\mathsf{F}_{\mathsf{int}}$, are aggregated by type and normalised by the total number of the intermolecular interactions in (A)-(C) respectively.

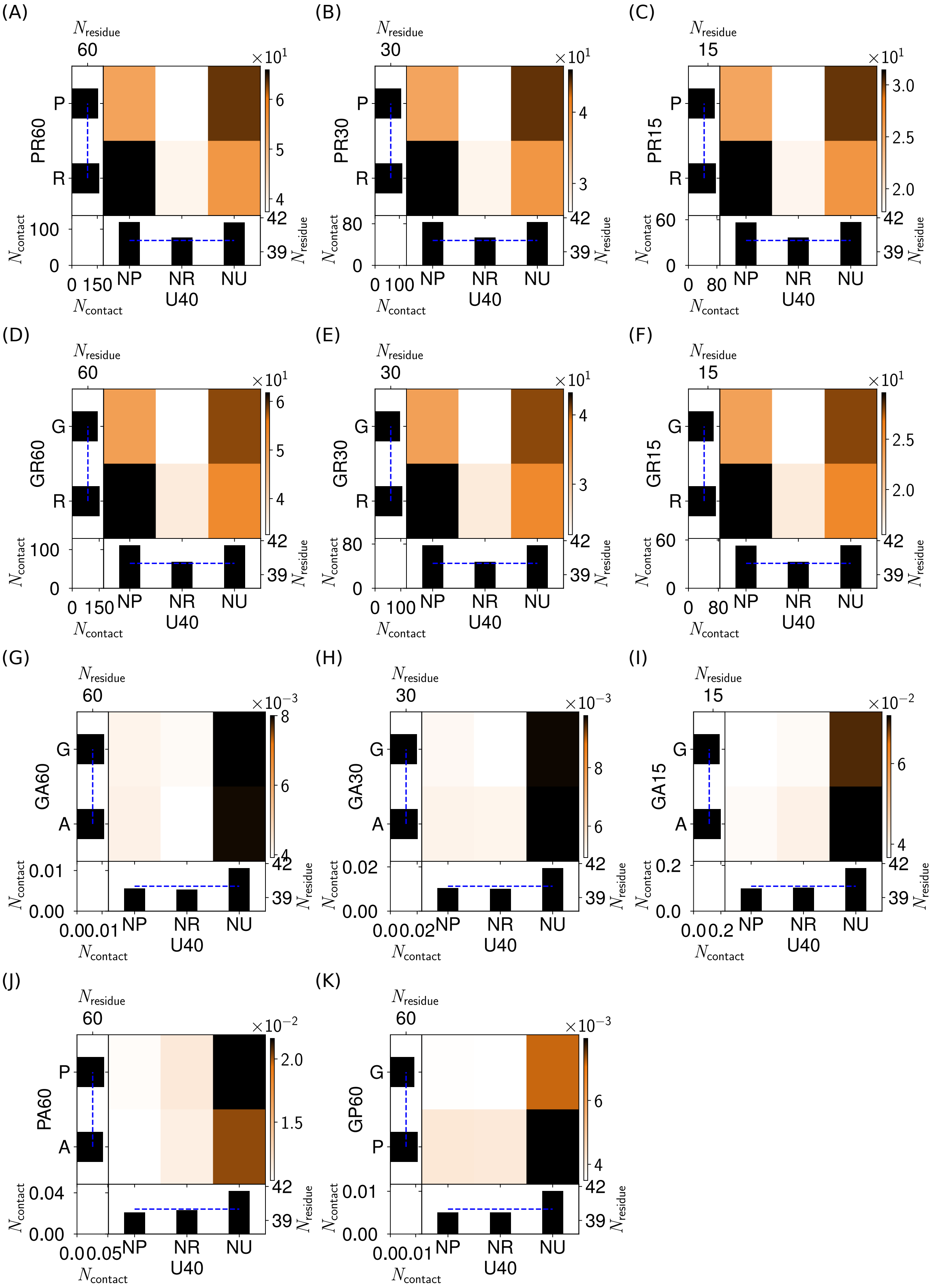

Figure S20: Intermolecular interaction summary for A) U40 with PR60 (50%, 50%) B) U40 with PR30 (50%, 50%) C) U40 with PR15 (50%, 50%) D) U40 with GR60 (50%, 50%) E) U40 with GR30 (50%, 50%) F) U40 with GR15 (50%, 50%) G) U40 with GA60 (50%, 50%) H) U40 with GA30 (50%, 50%) I) U40 with GA15 (50%, 50%) J) U40 with PA60 (50%, 50%) K) U40 with GP60 (50%, 50%) at 150 mM and 300 K.

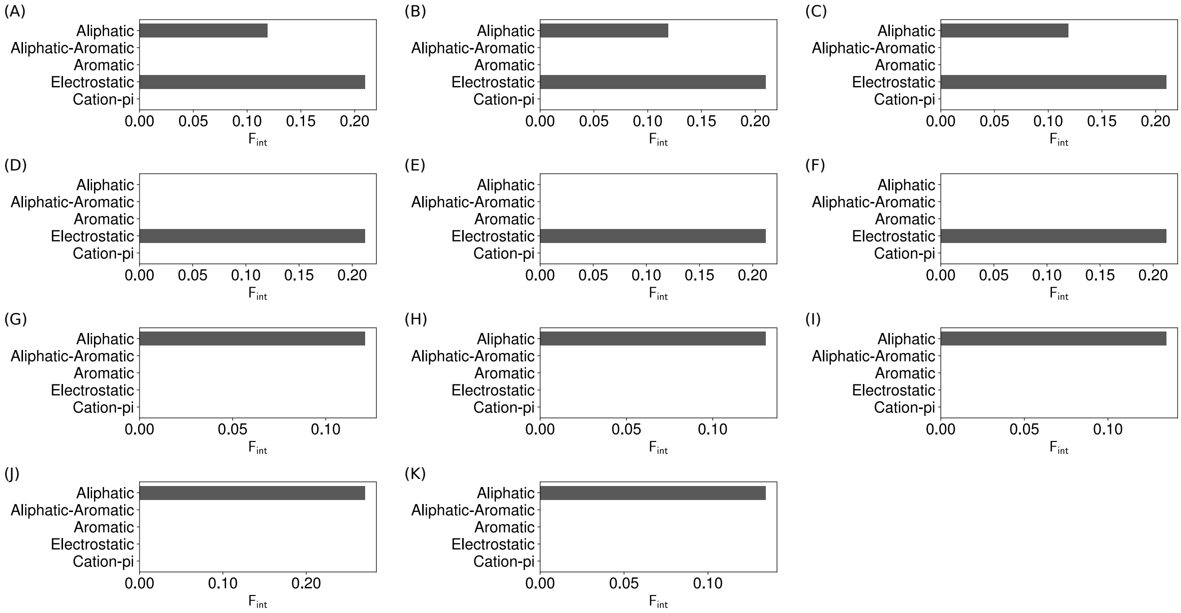

Figure S21: Intermolecular contact map by residue type for A) U40 with PR60 (50%, 50%) B) U40 with PR30 (50%, 50%) C) U40 with PR15 (50%, 50%) D) U40 with GR60 (50%, 50%) E) U40 with GR30 (50%, 50%) F) U40 with GR15 (50%, 50%) G) U40 with GA60 (50%, 50%) H) U40 with GA30 (50%, 50%) I) U40 with GA15 (50%, 50%) J) U40 with PA60 (50%, 50%) K) U40 with GP60 (50%, 50%) at 150 mM and 300 K.

8 Droplet simulation density data

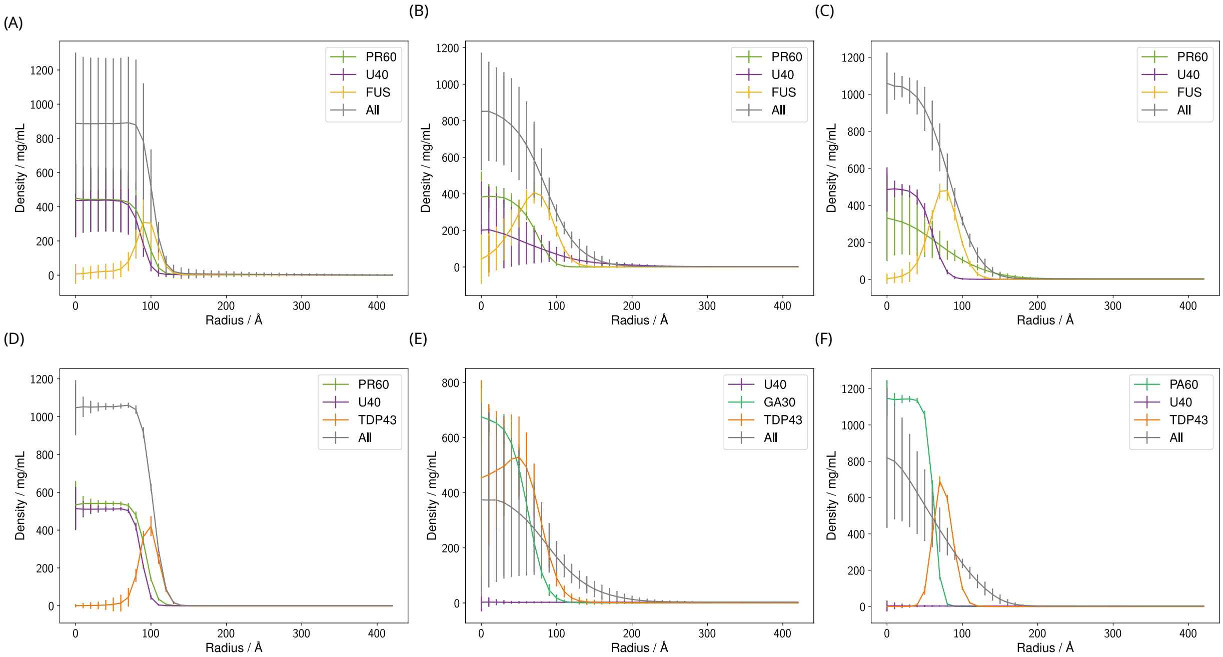

Figure S22: **Radial density information for a selection of ternary simulations:** (A) FUS-U_{40}-PR_{60} (33.3, 33.3, 33.3), (B) FUS-U_{40}-PR_{60} (37.5, 12.5, 50), (C) FUS-U_{40}-PR_{60} (37.5, 50, 12.5), (D) TDP43-U_{40}-PR_{60} (33.3, 33.3, 33.3), (E) TDP43-U_{40}-PA_{60} (33.3, 33.3, 33.3), (F) TDP43-U_{40}-GA_{30} (33.3, 33.3, 33.3).

9 System charge comparison

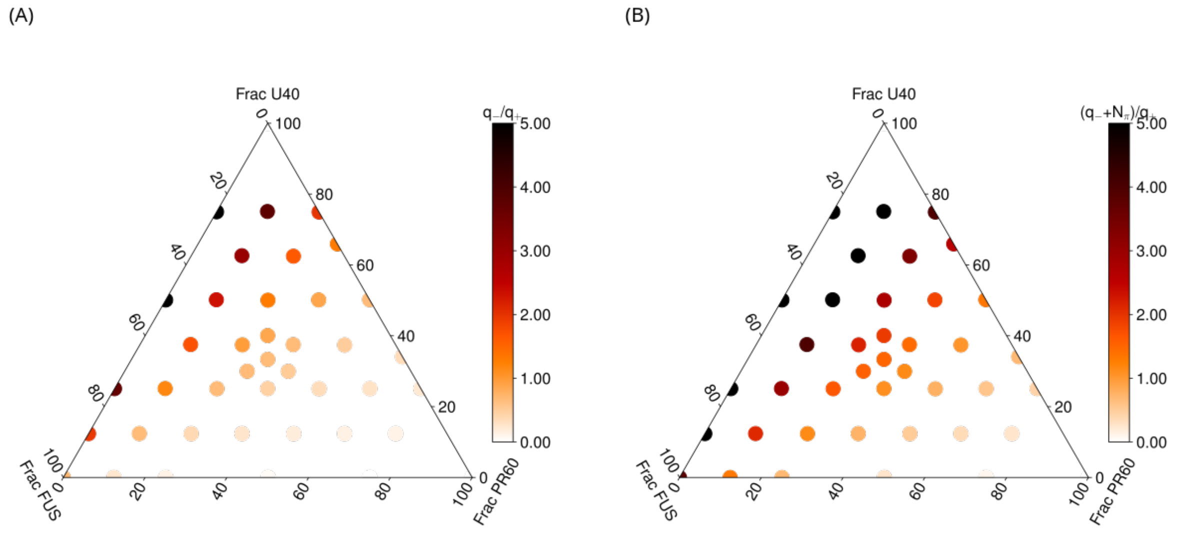

Figure S23: **Ternary phase diagrams showing ratio of sticker groups.** (A) q_-/q_+ (Charge ratio) (B) $q_-{+}N_\pi)/q_+$. Where q_-, q_+, N_π are the number of negative residues, the number of positive residues and the number of aromatic residues in the simulation.

References

[1] Ali Ghavami, Erik van der Giessen, and Patrick R. Onck. "Coarse-Grained Potentials for Local Interactions in Unfolded Proteins". In: *J. Chem. Theory Comput.* 9.1 (2013), pp. 432–440. ISSN: 1549-9618. DOI: 10.1021/ct300684j. URL: https://pubs.acs.org/doi/10.1021/ct300684j.

[2] Ali Ghavami et al. "Probing the Disordered Domain of the Nuclear Pore Complex through Coarse-Grained Molecular Dynamics Simulations". In: *Biophys. J.* 107.6 (2014), pp. 1393–1402. ISSN: 00063495. DOI: 10.1016/j.bpj.2014.07.060. URL: http://dx.doi.org/10.1016/j.bpj.2014.07.060%20https://linkinghub.elsevier.com/retrieve/pii/S000634951400808X.

[3] Ali Ghavami, Erik Van der Giessen, and Patrick R. Onck. "Sol–gel transition in solutions of FG-Nups of the nuclear pore complex". In: *Extreme Mech. Lett.* 22 (2018), pp. 36–41. ISSN: 23524316. DOI: 10.1016/j.eml.2018.04.006. URL: https://doi.org/10.1016/j.eml.2018.04.006.

[4] Philip Ketterer et al. "DNA origami scaffold for studying intrinsically disordered proteins of the nuclear pore complex". In: *Nat. Commun.* 9 (1 2018). ISSN: 20411723. DOI: 10.1038/s41467-018-03313-w.

[5] Ankur Mishra et al. "The effect of FG-nup phosphorylation on NPC selectivity: A one-bead-per-amino-acid molecular dynamics study". In: *Int. J. Mol. Sci.* 20 (3 2019). ISSN: 14220067. DOI: 10.3390/ijms20030596.

[6] Irina L. Rempel et al. "Age-dependent deterioration of nuclear pore assembly in mitotic cells decreases transport dynamics". In: *eLife* 8 (2019), pp. 1–26. ISSN: 2050084X. DOI: 10.7554/eLife.48186.

[7] Irina L. Rempel et al. "Flexible and Extended Linker Domains Support Efficient Targeting of Heh2 to the Inner Nuclear Membrane". In: *Structure* 28 (2 2020), 185–195.e5. ISSN: 18784186. DOI: 10.1016/j.str.2019.11.003.

[8] Hamidreza Jafarinia, Erik van der Giessen, and Patrick R. Onck. "Phase Separation of Toxic Dipeptide Repeat Proteins Related to C9orf72 ALS/FTD". In: *Biophys. J.* 119 (4 2020), pp. 843–851. ISSN: 00063495. DOI: 10.1016/j.bpj.2020.07.005. URL: https://linkinghub.elsevier.com/retrieve/pii/S0006349520305324.

[9] Alessio Fragasso et al. "Transport receptor occupancy in nuclear pore complex mimics". In: *Nano Res.* 15 (11 2022), pp. 9689–9703. ISSN: 19980000. DOI: 10.1007/s12274-022-4647-1.

[10] Alessio Fragasso et al. “A designer FG-Nup that reconstitutes the selective transport barrier of the nuclear pore complex”. In: *Nat. Commun.* 12.1 (2021), p. 2010. ISSN: 2041-1723. DOI: 10.1038/s41467-021-22293-y. URL: http://dx.doi.org/10.1038/s41467-021-22293-y%20http://www.nature.com/articles/s41467-021-22293-y.

[11] Hamidreza Jafarinia, Erik Van der Giessen, and Patrick R. Onck. “Molecular basis of C9orf72 poly-PR interference with the β-karyopherin family of nuclear transport receptors”. In: *Sci. Rep.* 12 (1 2022), p. 21324. ISSN: 2045-2322. DOI: 10.1038/s41598-022-25732-y. URL: https://www.nature.com/articles/s41598-022-25732-y.

[12] Maurice Dekker, Erik Van der Giessen, and Patrick R. Onck. “Phase separation of intrinsically disordered FG-Nups is driven by highly dynamic FG motifs”. In: *Proc. Natl. Acad. Sci. U. S. A.* 120 (25 2023). ISSN: 0027-8424. DOI: 10.1073/pnas.2221804120. URL: https://pnas.org/doi/10.1073/pnas.2221804120.

[13] Mark Driver and Patrick R. Onck. “Selective phase separation of transcription factors is driven by orthogonal molecular grammar”. In: *biorxiv* (2024). DOI: 10.1101/2024.04.12.589262.

[14] Andrés R. Tejedor et al. “‘RNA modulation of transport properties and stability in phase-separated condensates”. In: *Biophys. J.* 120 (23 2021), pp. 5169–5186. ISSN: 15420086. DOI: 10.1016/j.bpj.2021.11.003.

[15] G. Heesink et al. “Exploring Intra- and Inter-Regional Interactions in the IDP α-Synuclein Using smFRET and MD Simulations”. In: *Biomacromolecules* 24 (8 2023), pp. 3680–3688. ISSN: 15264602. DOI: 10.1021/acs.biomac.3c00404.

[16] L. Kind et al. “Structural properties of the HNF-1A transactivation domain”. In: *Front. Mol. Biosci.* 10 (2023). ISSN: 2296889X. DOI: 10.3389/fmolb.2023.1249939.

[17] Erik D. Holmstrom et al. “Disordered RNA chaperones can enhance nucleic acid folding via local charge screening”. In: *Nature Communications* 10 (1 2019), pp. 1–11. ISSN: 20411723. DOI: 10.1038/s41467-019-10356-0. URL: http://dx.doi.org/10.1038/s41467-019-10356-0.

[18] Lauren H. Kapcha and Peter J. Rossky. “A simple atomic-level hydrophobicity scale reveals protein interfacial structure”. In: *Journal of Molecular Biology* 426.2 (2014), pp. 484–498. ISSN: 10898638. DOI: 10.1016/j.jmb.2013.09.039. URL: http://dx.doi.org/10.1016/j.jmb.2013.09.039.
